# The impact of drought on the association between food security and mental health in a nationally representative Australian sample

**DOI:** 10.1186/1471-2458-14-1102

**Published:** 2014-10-24

**Authors:** Sharon Friel, Helen Berry, Huong Dinh, Léan O’Brien, Helen L Walls

**Affiliations:** Regulatory Institutions Network, The Australian National University, Canberra, Australia; National Centre for Epidemiology and Population Health, The Australian National University, Canberra, Australia; Faculty of Health, University of Canberra, Canberra, Australia; Faculty of Business, Government and Law, University of Canberra, Canberra, Australia; Leverhulme Centre for Integrative Research on Agriculture and Health, London, UK; London School of Hygiene and Tropical Medicine, London, UK

**Keywords:** Climate change, Drought, Food insecurity, Mental health, Urban, Rural, Australia

## Abstract

**Background:**

The association between food insecurity and mental health is established. Increasingly, associations between drought and mental health and drought and food insecurity have been observed in a number of countries. The impact of drought on the association between food insecurity and mental health has received little attention.

**Methods:**

Population-based study using data from a nationally representative panel survey of Australian adults in which participants report behaviour, health, social, economic and demographic information annually. Exposure to drought was modelled using annual rainfall data during Australia’s ‘Big Dry’. Regression modelling examined associations between drought and three indicative measures of food insecurity and mental health, controlling for confounding factors.

**Results:**

People who reported missing meals due to financial stress reported borderline moderate/high distress levels. People who consumed below-average levels of core foods reported more distress than those who consumed above the average level, while people consuming discretionary foods above the average level reported greater distress than those consuming below the threshold. In all drought exposure categories, people missing meals due to cost reported higher psychological distress than those not missing meals. Compared to drought-unadjusted psychological distress levels, in most drought categories, people consuming higher-than-average discretionary food levels reported higher levels of distress.

**Conclusions:**

Exposure to drought moderates the association between measures of food insecurity and psychological distress, generally increasing the distress level. Climate adaptation strategies that consider social, nutrition and health impacts are needed.

**Electronic supplementary material:**

The online version of this article (doi:10.1186/1471-2458-14-1102) contains supplementary material, which is available to authorized users.

## Background

Food security, nutritional status and mental health are strongly connected. Food insecurity, defined here as poor nutritional intake, insufficient amounts of food eaten and being unable to afford nutritious food is a problem affecting many households worldwide, including in Australia [1–5]. Food insecurity has been shown to be independently associated with higher levels of psychological distress, psychiatric disorders and poor child development [6–9].

Climate change is an important contemporary global health issue [10–14]. Internationally and in Australia, climate change is increasing food insecurity, with risks for nutrition and health. Through the increased frequency and duration of adverse weather events such as drought, cyclones and flooding, climate change can reduce agricultural productivity and the viability of agricultural support industries [15–18]. These climate impacts can affect food yields, household livelihoods and food prices, which influence dietary habits through food availability and affordability pathways [17, 19–21]. The 2001–2008 Australian drought, termed the ‘Big Dry’, and other extreme weather events are reported to have affected agricultural yields and food prices: between 2005 and 2007, vegetables in Australia increased in price by 33% and fruit by 43% [22].

Climate change is also having an impact on mental health [23–25]. Climate change can affect mental health directly by exposing people to the psychological trauma associated with higher frequency, intensity and duration of climate-related disasters [24], including extreme heat exposure [26], and also by harming landscapes, which diminishes the sense of belonging and solace that people derive from their connectedness to the land [27]. Indirectly, climate change may affect community wellbeing through damage to the economic and, consequently, the social fabric of communities [24, 25].

Recent Australian reports on the health consequences of climate change have identified an urgent need to understand how drought and long-term drying affect population health and wellbeing [10]. It is possible that exposure to drought may exacerbate the food and mental health relationship via potential negative impacts on the availability, livelihoods, food price and consumption of nutritious food, thereby creating acute and chronic stress.

It is likely that drought and long-term drying will exacerbate existing inequities in health risks [28]. Previous Australian analyses have shown that extreme drought is associated with psychological distress and rural/remote areas but not urban [29]. International and Australian evidence suggests that food security is socio-economically [2, 30, 31] and geographically distributed [32–42], with low-socioeconomic status and people living in rural areas more likely to report food insecurity.

**Figure 1:**
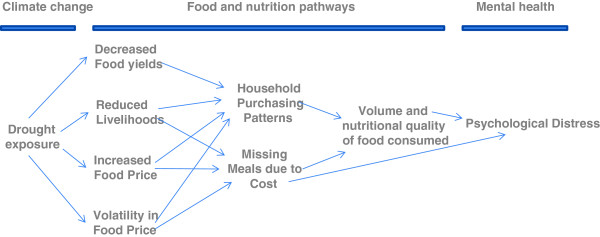
**Conceptualisation of food-related pathways from drought exposure to mental health.**

While the links between exposure to drought and food insecurity, and drought and mental health have been made in the literature, we are unaware of any studies that have explored the association between drought exposure, food security and mental health. This study will address this knowledge gap by examining the associations between indicative measures of food security and mental health in Australia during the last great drought, and exploring whether these associations are sensitive to levels of drought exposure across urban and rural locations.Figure 1 describes our hypothesized food-related pathways from drought exposure to mental health outcomes. These pathways are based on the existing literature and variables available in the main dataset used in the analysis. We hypothesize that the long dry in Australia has reduced local agricultural productivity and the viability of agricultural support industries. The knock-on effects of this has been, we hypothesize, a reduction in agricultural yields and therefore household income and increased food prices. These impacts have affected household purchasing patterns due to changes in the volume and types of food available for consumption and also the relative affordability of different foods. In some instances the impact on households’ income from the drought pressures will have meant missing meals due to cost. The impacts therefore on mental health arise from potential negative effects on food habits and nutrition, and from the acute and chronic stress of having to miss meals due to not having enough money (due to food price increases and/or reductions in household income).

## Methods

### Data sources

Our study is based on two existing sources of data: the Household, Income and Labour Dynamics in Australia (HILDA) Survey, which provides social, economic, demographic and health screening information about households in Australia annually since 2001, and monthly rainfall levels provided by the Australian Bureau of Meteorology.

#### HILDA data

The HILDA survey is a nationally representative panel survey of Australian adults aged 15 years and over in which participants complete interviewer-administered and self-complete questionnaires [43]. Data have been collected annually since the first Wave in 2001. Wave 1 included 13,969 people in 7,682 households, corresponding to a household response rate of 66% [44]. The HILDA Survey was initiated, and is funded by the Australian Government. Responsibility for the design and management of the survey is with the Melbourne Institute of Applied Economic and Social Research (University of Melbourne). The ANU obtained an Organisational Licence to use the HILDA data. In this study we used Wave 7, for which data were collected between August 2007 and February 2008, capturing a representative sample of the Australian population just before the end of the ‘Big Dry’, a period of extended extreme drought in Australia from early 2001 to mid-2008. Wave 7 contains measures of food insecurity and mental health.

#### Drought: Australian Bureau of Meteorology rainfall data

Drought indices were calculated using monthly rainfall data provided by the Australian Bureau of Meteorology (see *Variables* section below for more detail on the indices). Rainfall was gridded at a resolution of 0.25 degree of latitude-longitude for the period 1890–2008 for the whole of Australia. Barnes Scheme data interpolation techniques were used to ensure that there were no missing data for any part of the continent [45, 46].

#### Combining datasets

There were 5,012 respondents among the 12,789 people in Wave 7 who had not moved out their Census Collection Districts (CCDs) during the Big Dry (and thus who had not changed drought exposure) and who had participated in every wave of the study over the relevant years (2001–08), thus providing complete information. The unit records of these 5,012 respondents were linked to the drought data for the 712 CCDs in which they lived over that period. The final linked datasets are hereafter called ‘the data’.

### Variables

We included variables capturing four domains: (i) mental health, (ii) food insecurity, (iii) exposures to drought, and (iv) confounders.

#### Mental health

Mental health was assessed using the Kessler 10-item measure of general psychological distress (K10), which measures non-specific symptoms of anxiety and depression [47]. Possible K10 scores range from 10–50, with higher scores indicating greater distress [48]. Levels of distress were interpreted according to the Australian 2000 Health and Wellbeing Survey and the 2001 National Health Survey K10 cut-off scores (low distress 10–15, moderate distress 16–21, high distress 22–29 and very high distress 30–50) [49]. In the present study, the K10 mean score is 15.53 (*N = 5012, SE = 0.13)*, indicating, on average, low-moderate distress across the sample.

#### Food insecurity

We used three indicative measures of food insecurity. First, a single item in the HILDA Survey asks ‘Since the beginning of this year, did you go without meals because of a shortage of money?’ (yes/no). The two other measures were informed by the Australian Dietary Guidelines [50], which recommend people “enjoy a wide variety of nutritious foods from the five main (core) food groups every day” (grain (cereal) foods; vegetables and legumes; fruit; milk, yoghurt and cheese; and meat, fish, eggs, tofu, nuts, and legumes) and that they choose ‘discretionary’ foods only sometimes or in small amounts. Discretionary foods are foods not listed previously that are high in fats, salt and simple sugars.

The HILDA Survey (Wave 7) asks respondents to report the frequency of consumption of twelve food items (breads, legumes/pulses, pasta/rice, breakfast cereals, fish or shellfish, poultry, red meat, *biscuits/cakes, confectionary, snack foods, fried potatoes, processed meat*: discretionary foods in italics). We used these to create two new variables. Each food item was classified as a ‘core healthy’ or ‘highly-processed discretionary’ food. The response categories for frequency of food consumption were collapsed to create a dichotomous ‘regular consumption’ variable (yes = 1, no = 0) for each type of food, with ‘regular’ denoting foods consumed daily, twice or more per week or at least once per week. Responses were summed for each type of food and sample mean scores calculated. We used two categorisations: ‘lower-than-average regular consumption of core foods’ (yes/no) and ‘higher-than-average regular consumption of discretionary foods’ (yes/no).

#### Drought exposure

Using the annual rainfall data, Hutchinson Drought Indices were calculated for the 7-year period from 2001–02 (when the ‘Big Dry’ began) to 2007–08 (when participants were interviewed for the HILDA Survey, Wave 7, coinciding with the end of the drought) [51, 52]. Indices were calculated for CCDs using specialist software (PostgreSQL database with the PostGIS spatial extension) managed by the National Centre for Epidemiology and Population Health at the Australian National University.

Following the method developed by O’Brien et al. [53], these indices were used to identify five patterns of *relative* dryness, or ‘drought exposures’: (i) zero-to-moderate drought, (ii) very dry drought, (iii) recent long period of drought, (iv) constant drought, and (v) constant drought with a recent long period (Table 1). Associations have been found in rural and remote areas only between this last category of drought exposure and mental health [53]. The analyses in this paper therefore explicitly contrast urban and rural/remote experiences and use ‘constant and long dry’ as the reference category.

**Table 1:** **Drought categories based on cumulative annual rainfall**

Drought category	General description	Cumulative drought over 5 years	Cumulative drought over 7 years
Zero-to-moderate drought	Not exposed to extreme drought	-	-
Very dry drought	During drought the relative level of dryness is intensely dry	Population in top 19.8% of dryness	Population in top 15.9% of dryness
Recent long period	In relative dryness for long unbroken period in the last two years (five months of relative dryness elapse before Hutchinson count method begins)	In relative dryness for 14-to-27 months between 2003-2005/6	In relative dryness for 15-to-21 months between 2005-2007/8
Constant drought	In drought for an extreme number of months	In drought for 12-to-32 months between 2001-2005/6	In drought for 21-to-32 months between 2001-2007/8
Constant drought with a recent long period	Experienced both constant drought (i.e. many months) and a recent long period of relative dryness (i.e. unbroken dryness that developed into drought)	-	-

Note. The total number of months spent in drought was similar across the ‘constant’ drought and the ‘constant drought with recent long period’ categories.

#### Confounders

We controlled for the following potential confounding characteristics: (i) demographic (sex, age, relationship status, Aboriginal and Torres Strait Islander status); (ii) socio-economic status (education, employment status, equivalised household income, calculated using the Organisation for Economic Cooperation and Development modified equivalised-scale method) [54], and (iii) health-related behaviours (use of tobacco and alcohol, levels of physical activity).

There were up to 11% missing values for at least one variable in the dataset, which we imputed using a chained regression procedure, which is recognised as a suitable approach for imputing incomplete large, national and public datasets [55–57]. Sensitivity analyses showed that models using the dataset with imputation fitted the observed data better than those with missing data and were therefore used in all analyses. The results for complete cases only are available online in Additional file 1.

### Data analysis

The aim of this study is to examine whether the associations between forms of food insecurity and mental health in Australia are sensitive to levels of drought exposure across urban and rural locations. To do this we used four stages of analysis, with all analyses adjusted for confounding variables and for the complex multi-stage sampling design of the HILDA survey. We first tested the association between each of the three food insecurity items and psychological distress. The second and third stages of analysis explored associations between drought and psychological distress, and between drought and food insecurity items respectively. The final stage of analysis assessed the impact of drought on the food insecurity-psychological distress associations. For all analyses, the ‘constant and long dry’ category was used as the reference category because this was the category that showed a consistent statistically significant association between drought exposure and psychological distress and food insecurity.

*F*-tests and *Chi*-squared tests were employed to examine bivariate associations among variables. Regression analyses were used to estimate the mean values of psychological distress for each measure of food insecurity at different drought exposures. Three levels of significance are used in the paper, 10%, 5% and 1% because the sample is cross-sectional and is confined to participants who had not moved location during the ‘Big Dry’ period and hence a selected sub-sample of the study population. Given limited space, detailed regression analysis results are omitted but are available from the authors. Interaction terms between a dummy variable for urban residence and each drought category were included in the analyses to account for place-based differential impacts of drought. Two-level maximum likelihood regression analyses were used: the first level assumed fixed effects for confounders (demographic, socio-economic and health-related behaviours) while the second assumed random effects by CCD. The likelihood ratio test confirmed that multi-level regression was needed for each analysis, except for the regression analysis of food insecurity on drought. *P*-values are two-sided. Analyses were conducted using the STATA SE statistical software package version 12.

## Results

While almost half of the participants in HILDA Wave 7 experienced ‘zero to moderate’ drought exposure, 42.4% of people lived in areas that had experienced ‘very dry’ or ‘long dry’ conditions (Table 2). These experiences differed significantly between rural and urban areas. The proportion of people living in ‘constant dry’ and ‘constant and long dry’ did not differ significantly between urban and rural areas. The socio-demographic, socio-economic and health-related behaviours of people living in the different categories of drought exposure are shown in Table 3.

**Table 2:** **Summary characteristics of key indicators: drought exposure, food insecurity, and psychological distress, stratified by rural and urban location**

	Rural		Urban		All		Mean difference between rural and urban		
	***(N =919)***		***(N =4093)***		***(N =5012)***				
	%	N	%	N	%	N	%	SE	P-value
**Drought exposure**									
Zero and moderate	65.0	597	44.3	1814	47.3	2369	20.6	6.9	0.003
Very dry	5.5	50	13.4	550	12.3	616	-8.0	3.4	0.019
Long dry	18.1	166	32.1	1314	30.1	1509	-14.0	6.0	0.019
Constant dry	5.8	54	3.0	123	3.4	171	2.8	2.5	0.259
Constant and long dry	5.6	52	7.2	293	6.9	348	1.5	3.7	0.681
**Food insecurity**									
*Missing meals*									
Yes	1.8	16	1.6	63	1.6	79	0.2	0.6	0.707
No	98.2	903	98.4	4030	98.4	4933			
*Core food consumption*									
Below-average	32.8	301	31.6	1293	31.8	1592	1.2	2.4	0.635
Above-average	67.2	618	68.4	2800	68.2	3420			
*Discretionary food consumption*									
Above average	60.2	554	62.2	2545	61.9	3103	-2.0	2.7	0.466
Below average	39.8	365	37.8	1548	38.1	1909			
**Psychological distress,** ***Mean (SE)***									
	15.1	0.3	15.6	0.1	15.5	0.1	-0.5	0.3	0.104

**Table 3:** **Socio-demographic, socio-economic and health-related behaviours characteristics of respondents in each drought exposure category**

	Zero and moderate (N = 2450) N (%)	Very dry (N = 566) N (%)	Long dry (N = 1433) N (%)	Constant dry (N = 228) N (%)	Constant and long dry (N = 335) N (%)
Sex (% Male)	1120 (45.7)	241 (42.6)	668 (46.6)	108 (47.4)	151 (45.1)
Marital status (% Married/de facto)	1548 (63.2)	329 (58.1)	899 (62.7)	145 (63.6)	213 (63.6)
Age group (years) %					
15-25	342 (14.0)	104 (18.4)	221 (15.4)	34 (14.9)	49 (14.6)
26-39	178 (7.3)	40 (7.1)	124 (8.7)	18 (7.9)	39 (11.6)
40-55	821 (33.5)	160 (28.3)	481 (33.6)	80 (35.1)	106 (31.6)
56-65	479 (19.6)	113 (20.0)	242 (16.9)	37 (16.2)	67 (20.0)
+65	630 (26.4)	149 (26.3)	365 (25.5)	59 (25.9)	74 (22.2)
Indigeneity (% Indigenous Australians)	37 (1.5)	3 (0.5)	11 (0.8)	0 (0)	3 (0.9)
Equivalised Household Income (%)					
1st quintile	541 (22.0)	90 (15.9)	272 (19.0)	48 (21.1)	54 (16.1)
2nd quintile	548 (22.4)	95 (16.8)	253 (17.7)	46 (20.2)	58 (17.3)
3rd quintile	474 (19.4)	97 (17.1)	305 (21.3)	42 (18.4)	85 (25.4)
4th quintile	471 (19.2)	115 (20.3)	303 (21.1)	47 (20.6)	68 (20.3)
5th quintile	416 (17.0)	169 (29.9)	300 (21.0)	45 (19.7)	70 (20.9)
Employment status (% Employed)	1369 (55.9)	314 (55.5)	811 (56.6)	134 (58.8)	198 (59.1)
Education (% with at least year 12 or equivalent)	1418 (57.9)	381 (67.3)	892 (62.3)	141 (61.8)	215 (64.2)
Smoking status (% smokers)	910 (37.14)	236 (41.7)	652 (45.5)	92 (40.3)	148 (44.2)
Alcohol (% Moderate drinkers)	1220 (49.8)	290 (51.2)	718 (50.1)	117 (51.3)	170 (50.8)
Physical activity (% Active)	453 (18.5)	86 (15.2)	270 (18.8)	49 (21.5)	73 (21.8)

Relatively small numbers of people (1.6%) reported going without meals due to financial pressures (Table 2). Almost two-thirds consumed higher-than-average regular consumption of discretionary foods, regardless of location. The mean psychological distress score for the study participants was borderline none-to-moderate distress and did not differ between rural/urban locations.

### Food insecurity and psychological distress

Greater psychological distress was statistically significantly associated with each measure of food insecurity (Table 4). Those people who reported missing meals due to financial stress reported moderate-high distress levels. People who had lower-than-average regular consumption of core healthy foods reported more distress than others, while people with higher-than-average regular consumption of discretionary foods reported greater distress than those consuming below the threshold.

**Table 4:** **Mean levels of psychological distress by measures of food insecurity, adjusted for confounding variables**

Food insecurity		Mean score (SE)	Mean score difference (SE)	P-value
Missing meals	Yes	22.4 (0.6)	7.3 (0.6)	<0.001
	No	15.1 (0.1)		
Below-average consumption core food	Yes	15.5 (0.1)	0.4 (0.2)	0.009
	No	15.0 (0.1)		
Above-average consumption discretionary food	Yes	15.4 (0.1)	0.5 (0.2)	0.002
	No	14.9 (0.1)		

Note: Results for this analysis are presented for the whole sample only as the rural–urban interaction was not significant.

### Drought exposure and food insecurity

There was no statistically significant association found between drought exposure and missing meals in either rural or urban areas, except among the 5% of urban dwellers who experienced ‘constant dry’ who most often missed meals (Table 5; p <0.10). Rural people who had experienced ‘constant and long dry’ were more likely to regularly consume below-average levels of core foods than those experiencing ‘very dry’ (p <0.05) or ‘long dry’ (p <0.10)*.* Among urban participants, there were no significant associations between below-average core food consumption and drought exposures. While the proportions of people who consumed above-average discretionary food differed across drought categories, these differences were not statistically significant in either urban or rural areas.

**Table 5:** **Levels of food insecurity by type of drought exposure, stratified by urban and rural location, adjusted for confounding variables**

	Food insecurity								
	Missing meals			Below-average consumption core food			Above-average consumption discretionary food		
	Mean % (SE)	Mean difference (SE) ^1^	P-value	Mean % (SE)	Mean difference (SE) ^1^	P-value	Mean % (SE)	Mean difference (SE) ^1^	P-value
**Drought exposure (Rural)**									
Zero or Moderate	1.6 (0.01)	-0.1 (1.5)	0.934	33.2 (0.02)	-10.7 (7.5)	0.154	58.6 (0.03)	-0.5 (5.8)	0.927
Very Dry	n.a^2^	n.a^2^	n.a^2^	26.1 (0.03)	-17.8 (7.8)	0.024	70.4 (0.05)	11.3 (7.3)	0.121
Long Dry	2.3 (0.01)	0.6 (1.9)	0.744	31.0 (0.03)	-12.9 (7.6)	0.090	62.5 (0.04)	3.4 (6.4)	0.595
Constant Dry	3.9 (0.03)	2.2 (3.3)	0.511	27.5 (0.08)	-16.4 (10.1)	0.106	64.1 (0.07)	5.0 (8.9)	0.577
Constant and Long Dry	1.7 (0.02)	-	-	43.9 (0.07)	-	-	59.1 (0.05)	-	-
**Drought exposure (Urban)**									
Zero or Moderate	1.9 (0.01)	1.2 (0.8)	0.133	33.4 (0.02)	-0.8 (4.5)	0.857	64.7 (0.01)	4.3 (4.7)	0.366
Very Dry	0.9 (0.01)	0.1 (0.7)	0.925	29.4 (0.02)	-4.8 (4.7)	0.307	62.7 (0.03)	2.3 (5.4)	0.673
Long Dry	1.3 (0.00)	0.5 (0.7)	0.510	28.4 (0.02)	-5.8 (4.5)	0.191	59.4 (0.02)	-1.1 (4.8)	0.827
Constant Dry	4.7 (0.02)	3.9 (2.1)	0.062	41.5 (0.05)	7.3 (6.6)	0.272	56.5 (0.03)	-4.0 (5.3)	0.455
Constant and Long Dry	0.8 (0.01)	-	-	34.3 (0.04)	-	-	60.4 (0.05)	-	-

^1^The mean score difference measures the difference in the estimated mean psychological distress score between ‘constant and long dry’ and each other drought category.

^2^The ‘very dry’ drought category is dropped in the regression of missing meals because this variable perfectly predict the failure (missing meals =0), causing this variable’s coefficient to be unidentified.

### Drought exposure and psychological distress

In rural areas, people exposed to ‘constant and long dry’ drought reported a moderate level of distress while those living in any other category reported little distress (Table 6). In urban areas, people reported a low level of distress for all drought categories, with urban dwellers exposed to ‘constant and long dry’ reporting the lowest level of psychological distress compared with other categories.

**Table 6:** **Levels of psychological distress by type of drought exposure, stratified by rural and urban location, adjusted for confounding variables**

	Psychological distress					
	Rural			Urban		
Drought exposure	Mean score (SE)	Mean score difference ^1^ (SE)	P-value	Mean score (SE)	Mean score difference ^1^ (SE)	P-value
Zero or Moderate	15.0 (0.3)	-2.8 (1.1)	0.009	15.4 (0.1)	0.6 (0.4)	0.087
Very Dry	15.0 (0.8)	-2.8 (1.3)	0.029	15.2 (0.3)	0.4 (0.4)	0.325
Long Dry	15.1 (0.5)	-2.7 (1.2)	0.019	15.0 (0.2)	0.38 (0.4)	0.451
Constant Dry	15.5 (0.7)	-2.3 (1.3)	0.078	14.9 (0.5)	0.2 (0.6)	0.805
Constant and Long Dry	17.8 (1.1)	-	-	14.8 (0.3)	-	-

^1^The mean score difference measures the difference in the estimated mean psychological distress score between ‘constant and long dry’ and each other drought category.

### Drought, food insecurity and psychological distress

Table 7 presents the mean psychological distress scores for each food insecurity item by category of drought exposure. In all categories of drought exposure, people missing meals reported greater psychological distress than other participants. This association was statistically significant for all drought exposures except ‘constant and long dry’. Compared to mean unadjusted distress scores, people experiencing ‘very dry’ drought reported a significantly higher level of distress while people living in ‘constant and long dry’ reported the lowest.

**Table 7:** **Mean psychological distress scores in different measures of food insecurity, according to level of drought exposure**

Food insecurity indicators	Drought exposures										Test for difference across drought exposures	
	Zero or moderate		Very dry		Long dry		Constant dry		Constant and long dry			
	Mean (SE)	P-value	Mean (SE)	P-value	Mean (SE)	P-value	Mean (SE)	P-value	Mean (SE)	P-value	Chi ^2^ (4)	P-value
**Missing meals**												
Yes	21.7 (0.8)		31.7 (2.4)		23.2 (1.2)		19.7 (2.0)		18.4 (2.7)		18.4	0.001
No	15.2 (0.1)		15.0 (0.2)		15.0 (0.2)		14.8 (0.4)		14.9 (0.3)			
Difference	6.5 (0.8)	<0.001	16.6 (2.4)	<0.001	8.2 (1.2)	<0.001	4.9 (2.0)	0.015	3.5 (2.7)	0.187		
**Below-average consumption core food**												
Yes	15.6 (0.2)		15.5 (0.4)		15.4 (0.3)		14.9 (0.6)		15.4 (0.5)		1.1	0.894
No	15.2 (0.1)		15.1 (0.3)		14.9 (0.2)		14.9 (0.5)		14.7 (0.4)			
Difference	0.4 (0.2)	0.054	0.4 (0.5)	0.384	0.5 (0.3)	0.152	0.0 (0.7)	0.983	0.7 (0.6)	0.329		
**Above-average consumption discretionary food**												
Yes	15.5 (0.2)		15.6 (0.3)		15.2 (0.2)		14.7 (0.5)		15.5 (0.4)		2.12	0.714
No	15.0 (0.2)		14.6 (0.4)		14.8 (0.2)		15.2 (0.6)		14.2 (0.5)			
Difference	0.5 (0.2)	0.050	1.0 (0.5)	0.036	0.4 (0.3)	0.178	-0.5 (0.7)	0.465	1.3 (0.6)	0.033		

Note: Results are presented for the whole sample only as the rural–urban interaction was not significant in this analysis.

All analyses are adjusted for confounding variables.

A somewhat different pattern was observed for core foods consumption. Although people with below-average consumption of core foods reported greater distress than those consuming above the average, this association was only statistically significant among people experiencing ‘zero and moderate dry’ drought.

For discretionary food consumption, significantly higher levels of distress were observed in people experiencing ‘zero or moderate’, ‘very dry’, and ‘constant and long dry’ drought. In each of these drought categories, people consuming above-average levels of discretionary foods reported a higher level of distress than those consuming below-average levels.

The level of psychological distress among people who reported missing meals differed significantly across categories of drought exposure. The levels of psychological distress did not differ significantly across categories of drought exposure for the two other measures of food insecurity.

## Discussion

We describe the findings of a large population-based study examining the associations between drought, food insecurity and people’s mental health in urban and rural settings in Australia.

Our findings support the strong evidence from other settings that food insecurity is associated with psychological distress [6–9]. Study participants who reported missing meals, or consumed below-average levels of core foods or above-average levels of discretionary foods reported moderate-high distress levels.

The findings also shed further light on the emerging international evidence regarding the relationship between climate change and food insecurity (increased during drought), and between climate change and mental health (harmed by weather-related disasters such as drought). Our main observation was the significant association between missing meals and exposure to drought, with urban dwellers who lived in ‘constant dry’ weather conditions more likely to miss meals due to financial pressures. This pattern was similar among rural dwellers but was not statistically significant, probably due to small numbers. Similarly, our study substantiates some of the conceptual analysis linking climate change and mental health [24, 26]: rural people who had been exposed to ‘constant and long dry’ drought reported a moderate level of distress while those living in any other category reported little distress. In urban areas, where there may be less disadvantage and more access to products and services, people reported a low level of distress for all drought categories.

We have shown that exposure to drought affects the food and mental health association, possibly by reducing the availability, affordability and consumption of nutritious food, which are each linked to increasing mental health problems [58, 59]. However, the relationship is not straightforward - the type of drought experienced can have positive or negative effects. Drought appears to moderate the association between food insecurity and mental health in the following ways:

*First*, compared to the average unadjusted distress score, people experiencing ‘very dry’ drought reported a significantly higher level of distress whereas people living in ‘constant and long dry’ drought reported the lowest. These findings suggest that living in constant and long dry drought affects the association between missing meals and mental health. We can hypothesize that when drought is a predictable outcome, households have some capacity to plan expenditures thereby preventing them from being caught short financially. In addition, persons who choose to remain in long term drought areas may have financial reserves to buffer their consumption against unexpected price and income changes. In contrast, in areas where drought occurs but usually not every year, households may spend based on “normal” income or prices for the locale and the onset of drought may come as a surprise.

*Second*, people consuming below-average levels of core foods reported greater distress than those consuming above-average levels, this association is only statistically significant among people experiencing ‘zero and moderate dry’ drought. This suggests that being in some kind of extreme drought disrupts the association between core food consumption and psychological distress that is observed in times of relatively normal weather conditions. While we do not have data to empirically explore the reasons for this, it may be that hunger is suppressed due to the heat associated with drought.

*Third,* compared to the average unadjusted distress score, significantly higher levels of distress were observed in people who consumed above-average levels of discretionary food among those who experienced ‘zero or moderate’, ‘very dry’, and ‘constant and long dry’ drought. The magnitude of the mean difference for the two drought categories are twice as big as the others, suggesting that these type of drought experiences amplified people’s distress by increasing people’s consumption of discretionary unhealthy food.

These relationships are not simple or straightforward. There are many interconnected factors that affect food consumption and mental health, including the social and built environments in which people live [60]. While such factors may or may not be directly related to drought, they will moderate and mediate its impact. Additionally, we do not know from these data whether the food insecurity effects of drought are through its impact on local food prices or through the impact of drought on incomes earned by persons in the affected area, or if food insecurity is an outcome of chronically insufficient income, or due to high volatility of purchasing power of income. The nature and scope of the cross-sectional data does not allow us to interrogate the complexity of these relationships and warrants further investigation.

Limitations of our study reduce the generalisability of our findings. First, we selected participants (~2/5 total sample) who did not move in the period measured by the drought variable (2001–2007). Disadvantaged Australians tend to move house more often [61], are more likely to experience poor mental health, and are much more often lost to follow-up than other respondents and were thus under-represented in the final datasets [62, 63]. This may explain why the observed levels of going without meals due to financial stress are lower than those reported in other Australian studies [2, 30]. Australia is a vast, sparsely populated continent in which the large majority lives on the seaboard, where most of the rain occurs. As the drought definitions used in this study rely on precipitation deviations from long-term norms, greater deviations will be found where precipitation is greater (i.e. where there is more variance from which to deviate). Thus, it is less likely that rural and remote areas will register as deviating significantly from their norms (because they tend to have less precipitation in the first place) and they will – incorrectly – appear to have lower rates of experiencing drought. Our study thus likely underestimates the prevalence and impact of drought in these areas. In addition, the HILDA Survey does not sample from remote and very remote locations.

Another limitation of our study is the use of three indicative measures of food insecurity. Ideally we would have used internationally validated food security assessment scales. However, as this was a secondary analysis of an existing dataset, we were constrained by the food measures recorded in the dataset. Finally, the analysis is based on self-reported food insecurity and mental health, likely causing poor mental health to be underreported [64, 65], and indicating underestimation of associations found. Despite these limitations, we conducted, to our knowledge, the first nationally representative study examining modelled drought conditions, food insecurity and mental health. We have shown that some types of drought can be harmful to mental health because they increase food insecurity and that these circumstances tend to be more common in rural areas.

## Conclusions

Drought is an increasing public health problem globally, likely to exacerbate existing health risks and conditions. The impact of drought on the well-established and strong association between food insecurity and mental health has received little attention. In this study, exposure to Australia’s ‘Big Dry’ moderates the association between measures of food insecurity and psychological distress, generally increasing the distress level. Climate adaptation strategies that consider social, nutrition and mental health impacts are needed.

## Electronic supplementary material

Additional file 1: **Analysis without imputation.** (PDF 290 KB)
