# Supplementary material for: The impact of drought on the association between food security and mental health in a nationally representative Australian sample
Source: BMC Public Health. 2014 Oct 24;14:1102. doi: 10.1186/1471-2458-14-1102 (PMC4288639; doi:10.1186/1471-2458-14-1102)
Supplement: Supplementary file 1 — Additional file 1: Analysis without imputation. (PDF 290 KB) [file 12889_2013_7365_MOESM1_ESM.pdf]

Analysis without imputation

Table 2a Summary characteristics of key indicators: drought exposure, food insecurity, and psychological distress, stratified by rural and urban location

	**Rural** *(N=919)*[a]		**Urban** *(N=4093)*[a]		**All** *(N=5012)*		**Mean difference between rural and urban**		
	**%**	**N**	**%**	**N**	**%**	**N**	**%**	**SE**	**P-value**
**Drought exposure**									
Zero and Moderate	65.0	597	44.3	1814	47.3	2369	20.6	6.9	0.003
Very Dry	5.5	50	13.4	550	12.3	616	-8.0	3.4	0.019
Long Dry	18.1	166	32.1	1314	30.1	1509	-14.0	6.0	0.019
Constant Dry	5.8	54	3.0	123	3.4	171	2.8	2.5	0.259
Constant and Long Dry	5.6	52	7.2	293	6.9	348	1.5	3.7	0.681
**Food insecurity**[a]									
Missing meals									
Yes	1.8	16	1.5	60	1.6	78	0.2	0.7	0.663
No	98.2	882	98.4	3941	98.4	4821			
Core food consumption									
Below-average	33.1	292	31.4	1234	31.7	1526	1.7	2.4	0.635
Above-average	66.9	591	68.6	2696	68.3	3287			
Discretionary food consumption									
Above average	60.0	540	62.4	2470	62.1	3017	-2.0	2.7	0.466
Below average	40.0	360	37.6	1489	37.9	1842			
**Psychological distress,** ***Mean (SE)***									
	15.1	0.3	15.6	0.1	15.5	0.1	-0.5	0.3	0.104

Note: [a] Because of missing values, the sample sizes for food insecurity are variable

Table 4a Mean levels of psychological distress by measures of food insecurity, adjusted for confounding variables

Food insecurity		Mean Score (SE)	Mean score difference (SE)	P-value
Missing meals	Yes	23.2 (0.6)	8.3 (0.6)	<0.001
	No	14.9 (0.1)		
Below-average consumption core food	Yes	15.5 (0.1)	0.5 (0.2)	0.004
	No	15.0 (0.1)		
Above-average consumption discretionary food	Yes	15.3 (0.1)	0.5 (0.2)	0.001
	No	14.7 (0.1)		

Note: Results for this analysis are presented for the whole sample only as the rural-urban interaction was not significant

Table 5a Levels of food insecurity by type of drought exposure, stratified by urban and rural location, adjusted for confounding variables

	Food insecurity								
	Missing meals			Below-average consumption core food			Above-average consumption discretionary food		
	Mean % (SE)	Mean difference (SE)[1]	P-value	Mean % (SE)	Mean difference (SE)[1]	P-value	Mean % (SE)	Mean difference (SE)[1]	P-value
**Drought exposure (Rural)**									
Zero or Moderate	1.6 (0.5)	-0.1 (1.5)	0.934	33.2 (2.4)	-10.7 (7.5)	0.154	58.6 (3.0)	-0.5 (5.8)	0.927
Very Dry	n.a[2]	n.a[2]	n.a[2]	26.1 (3.3)	-17.8 (7.8)	0.024	70.4 (5.0)	11.3 (7.3)	0.121
Long Dry	2.3 (0.7)	0.6 (1.9)	0.744	31.0 (3.2)	-12.9 (7.6)	0.090	62.5 (4.1)	3.4 (6.4)	0.595
Constant Dry	3.9 (2.8)	2.2 (3.3)	0.511	27.5 (7.6)	-16.4 (10.1)	0.106	64.1 (7.4)	5.0 (8.9)	0.577
Constant and Long Dry	1.7 (1.6)	-	-	43.9 (7.1)	-	-	59.1 (5.0)	-	-
**Drought exposure (Urban)**									
Zero or Moderate	1.9 (0.5)	1.2 (0.8)	0.133	33.4 (1.9)	-0.8 (4.5)	0.857	64.7 (1.3)	4.3 (4.7)	0.366
Very Dry	0.9 (0.8)	0.1 (0.7)	0.925	29.4 (2.2)	-4.8 (4.7)	0.307	62.7 (2.7)	2.3 (5.4)	0.673
Long Dry	1.3 (0.4)	0.5 (0.7)	0.510	28.4 (1.7)	-5.8 (4.5)	0.191	59.4 (1.5)	-1.1 (4.8)	0.827
Constant Dry	4.7 (2.0)	3.9 (2.1)	0.062	41.5 (5.2)	7.3 (6.6)	0.272	56.5 (2.7)	-4.0 (5.3)	0.455
Constant and Long Dry	0.8 (0.6)	-	-	34.3 (4.1)	-	-	60.4 (4.6)	-	-

[1]The mean score difference measures the difference in the estimated mean psychological distress score between 'constant and long dry' and each other drought category

[2]The 'very dry' drought category is dropped in the regression of missing meals because this variable perfectly predict the failure (missing meals = 0), causing this variable's coefficient to be unidentified.

Table 6a Levels of psychological distress by type of drought exposure, stratified by rural and urban location, adjusted for confounding variables

	Psychological distress					
	Rural			Urban		
Drought exposure	Mean score (SE)	Mean score difference[1] (SE)	P-value	Mean score (SE)	Mean score difference[1] (SE)	P-value
Zero or Moderate	14.8 (0.2)	-3.4 (1.1)	0.002	15.3 (0.1)	0.6 (0.4)	0.079
Very Dry	14.9 (0.7)	-3.3 (1.3)	0.009	15.1 (0.3)	0.4 (0.4)	0.390
Long Dry	14.8 (0.5)	-3.4 (1.1)	0.003	15.0 (0.2)	0.3 (0.4)	0.481
Constant Dry	15.5 (0.7)	-2.7 (1.3)	0.034	14.9 (0.5)	0.2 (0.6)	0.784
Constant and Long Dry	18.8 (1.0)	-	-	14.7 (0.3)	-	-

[1]The mean score difference measures the difference in the estimated mean psychological distress score between ‘constant and long dry’ and each other drought category

Table 7a Mean psychological distress scores in different measures of food insecurity, according to level of drought exposure

Food insecurity indicators	Drought exposures									
	Zero or Moderate		Very Dry		Long Dry		Constant Dry		Constant and Long Dry	
	**Mean (SE)**	**P-value**	**Mean (SE)**	**P-value**	**Mean (SE)**	**P-value**	**Mean (SE)**	**P-value**	**Mean (SE)**	**P-value**
**Missing meals**										
Yes	22.3 (0.8)		31.9 (2.3)		24.3 (1.2)		19.7 (2.0)		18.4 (3.7)	
No	15.0 (0.1)		14.9 (0.2)		14.8 (0.2)		14.5 (0.4)		14.9 (0.3)	
Yes/No difference	7.3 (0.8)	<0.001	17.0 (2.3)	<0.001	9.5 (1.2)	<0.001	5.2 (2.0)	0.009	7.2 (3.7)	0.053
**Below-average consumption core food**										
Yes	15.5 (0.2)		15.5 (0.4)		15.5 (0.3)		14.9 (0.6)		15.1 (0.5)	
No	15.1 (0.1)		15.0 (0.3)		14.8 (0.2)		14.9 (0.5)		14.7 (0.4)	
Yes/No difference	0.5 (0.2)	0.062	0.5 (0.5)	0.356	0.7 (0.3)	0.030	-0.0 (0.7)	0.959	0.4 (0.6)	0.570
**Above-average consumption discretionary food**										
Yes	15.4 (0.2)		15.4 (0.3)		15.1 (0.2)		14.6 (0.5)		15.5 (0.4)	
No	14.9 (0.2)		14.6 (0.4)		14.7 (0.2)		15.0 (0.6)		14.0 (0.5)	
Yes/No difference	0.5 (0.2)	0.037	0.8 (0.5)	0.072	0.4 (0.3)	0.145	-0.5 (0.7)	0.525	1.5 (0.6)	0.014

Note: Results are presented for the whole sample only as the rural-urban interaction was not significant in this analysis. All analyses are adjusted for confounding variables.
